# Supplementary material for: Synthesis and In Vitro Photodynamic Activity of Cationic Boron Dipyrromethene-Based Photosensitizers Against Methicillin-Resistant Staphylococcus aureus
Source: Biomedicines. 2020 May 29;8(6):140. doi: 10.3390/biomedicines8060140 (PMC7344895; doi:10.3390/biomedicines8060140)
Supplement: Supplementary file 1 [file biomedicines-08-00140-s001.pdf]

Supplementary Materials

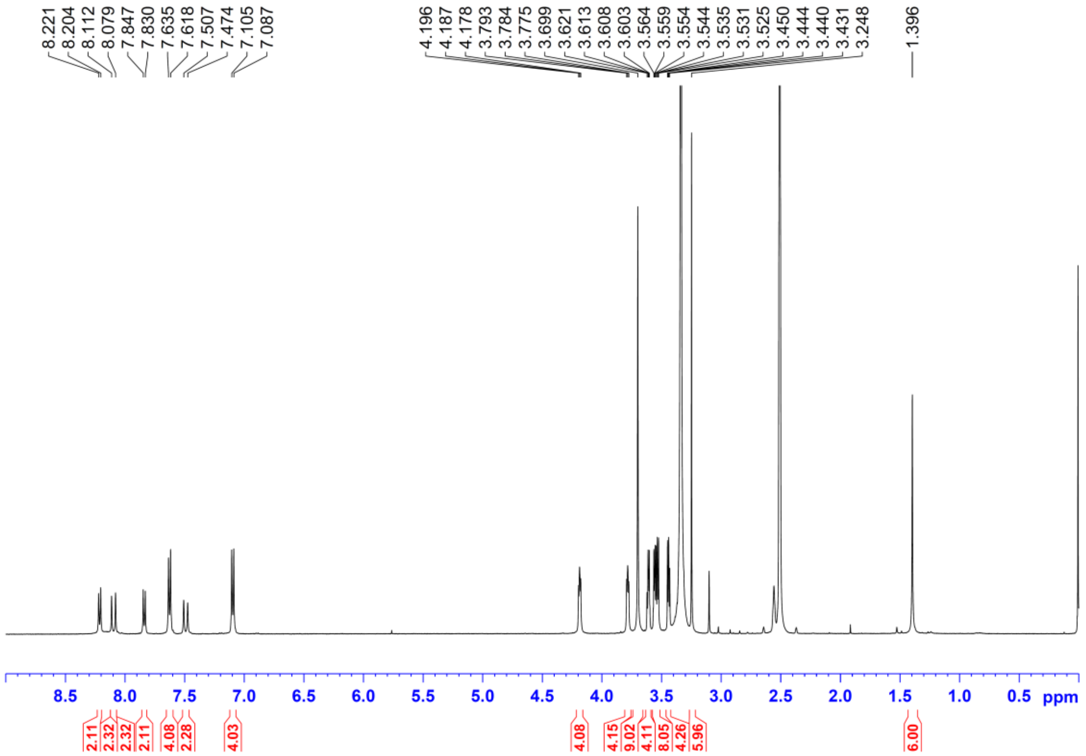

(**a**)

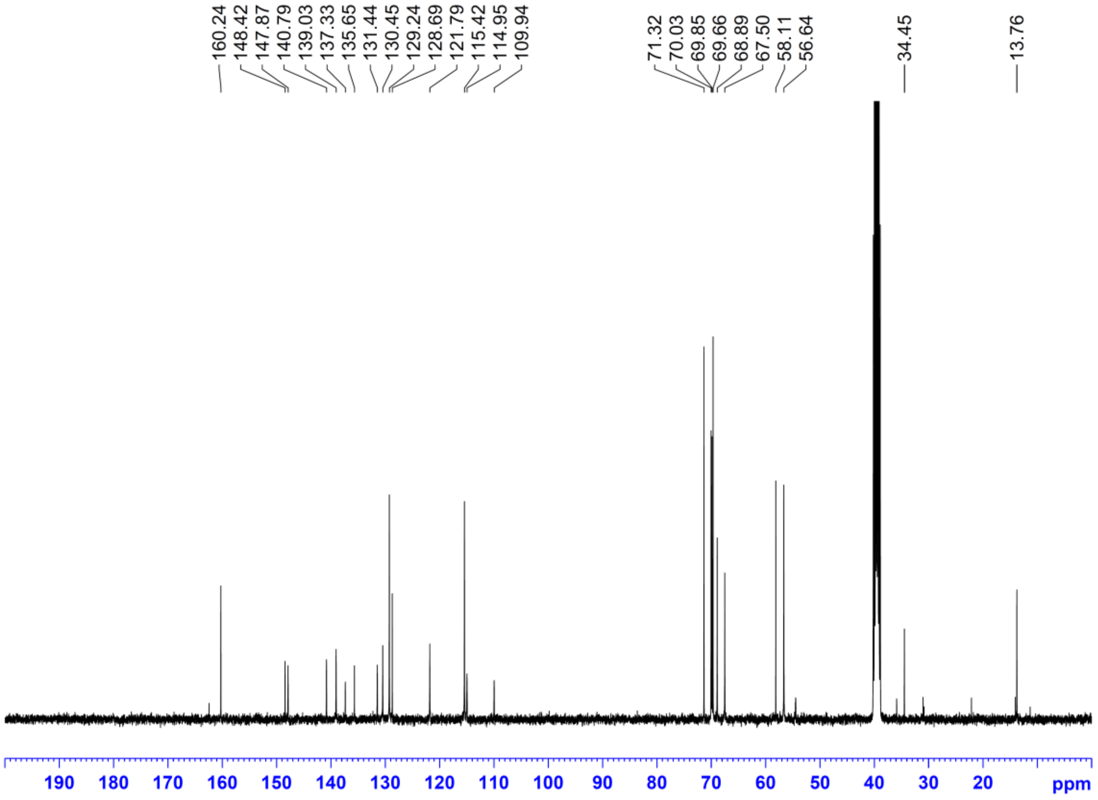

(**b**)

Figure S1. (**a**) ^{1}H and (**b**) $^{13}C\{^{1}H\}$ NMR spectra of 1 in DMSO-d_6.

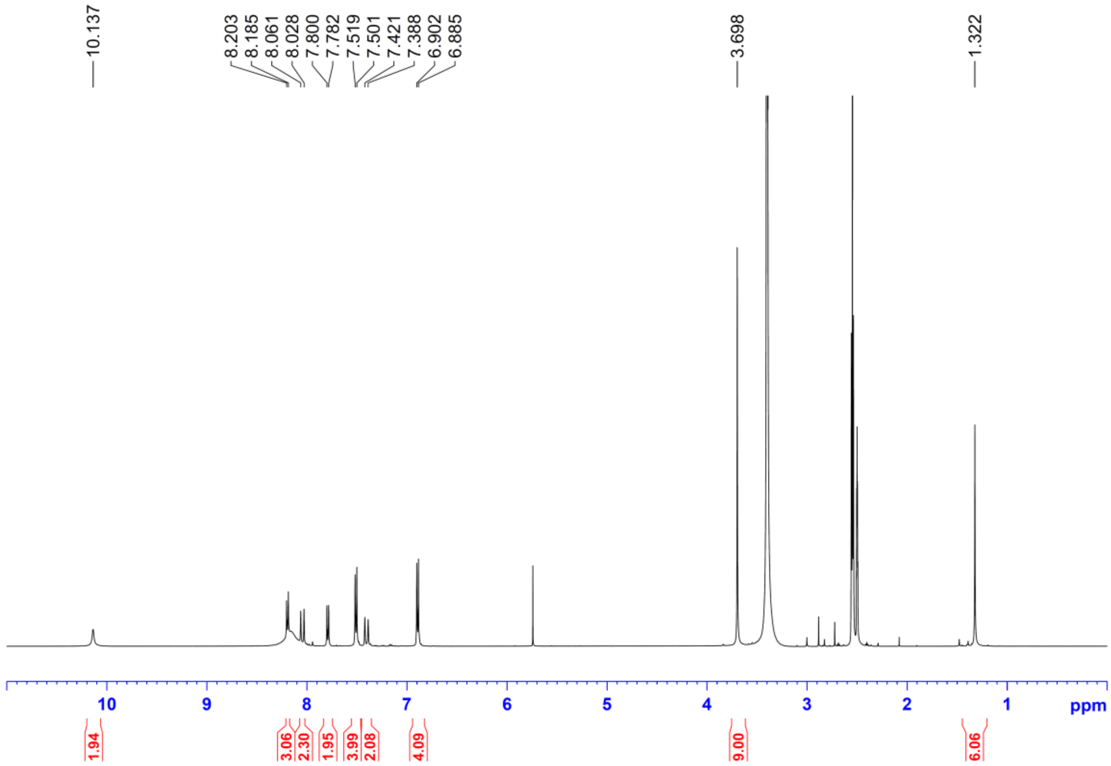

(**a**)

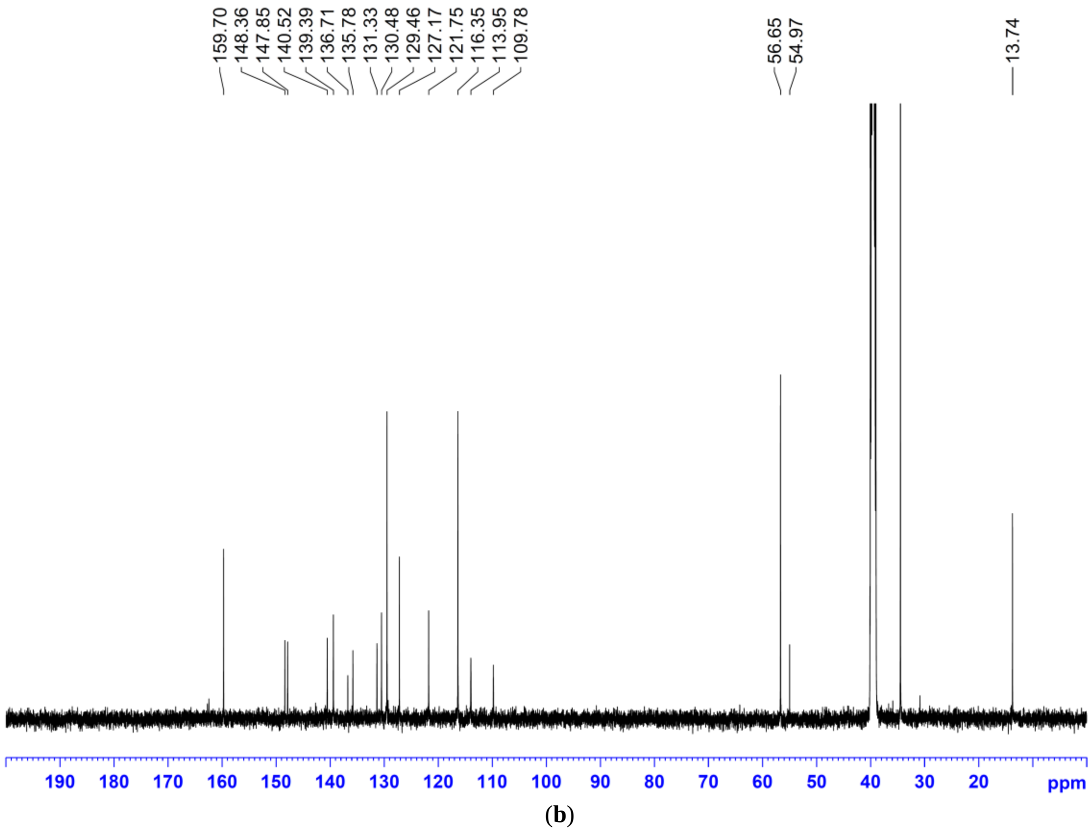

(**b**)

Figure S2. (**a**) ^{1}H and (**b**) $^{13}C\{^{1}H\}$ NMR spectra of 2 in DMSO-d_6.

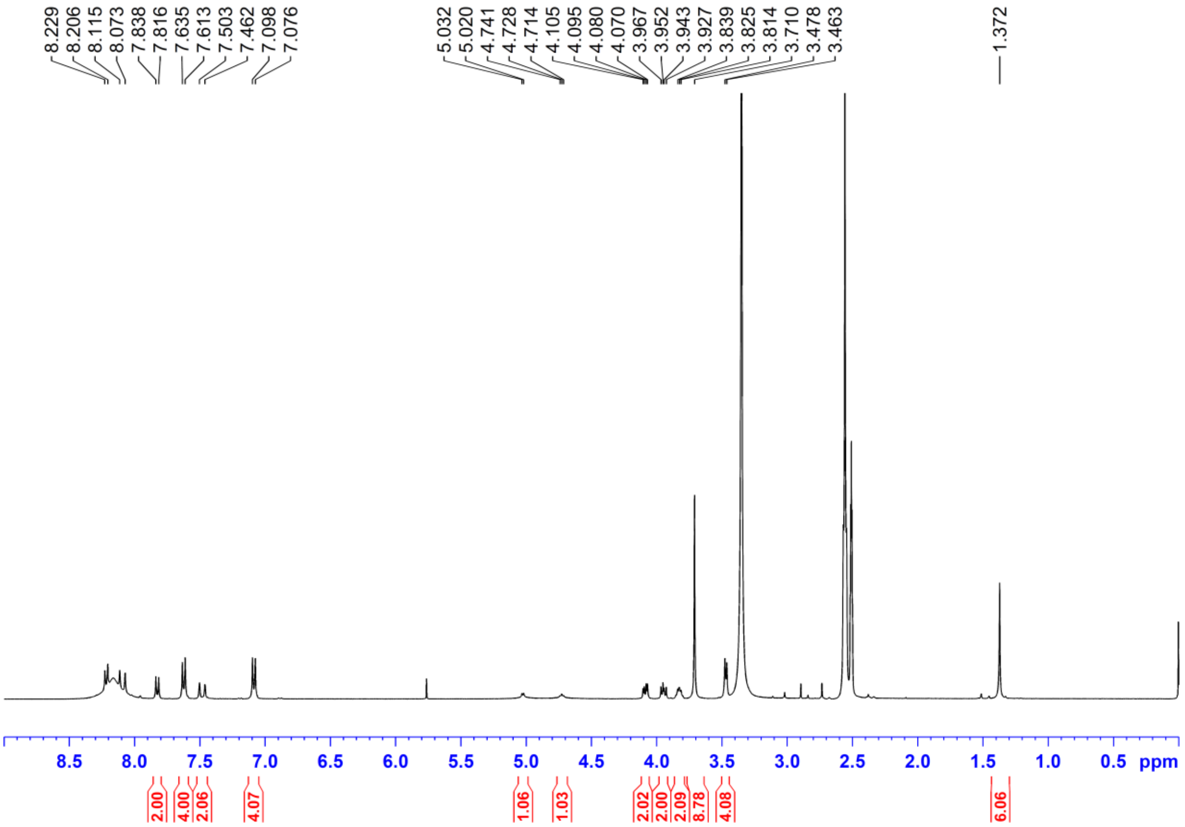

Figure S3. ^1H NMR spectrum of 3 in DMSO-d_6.

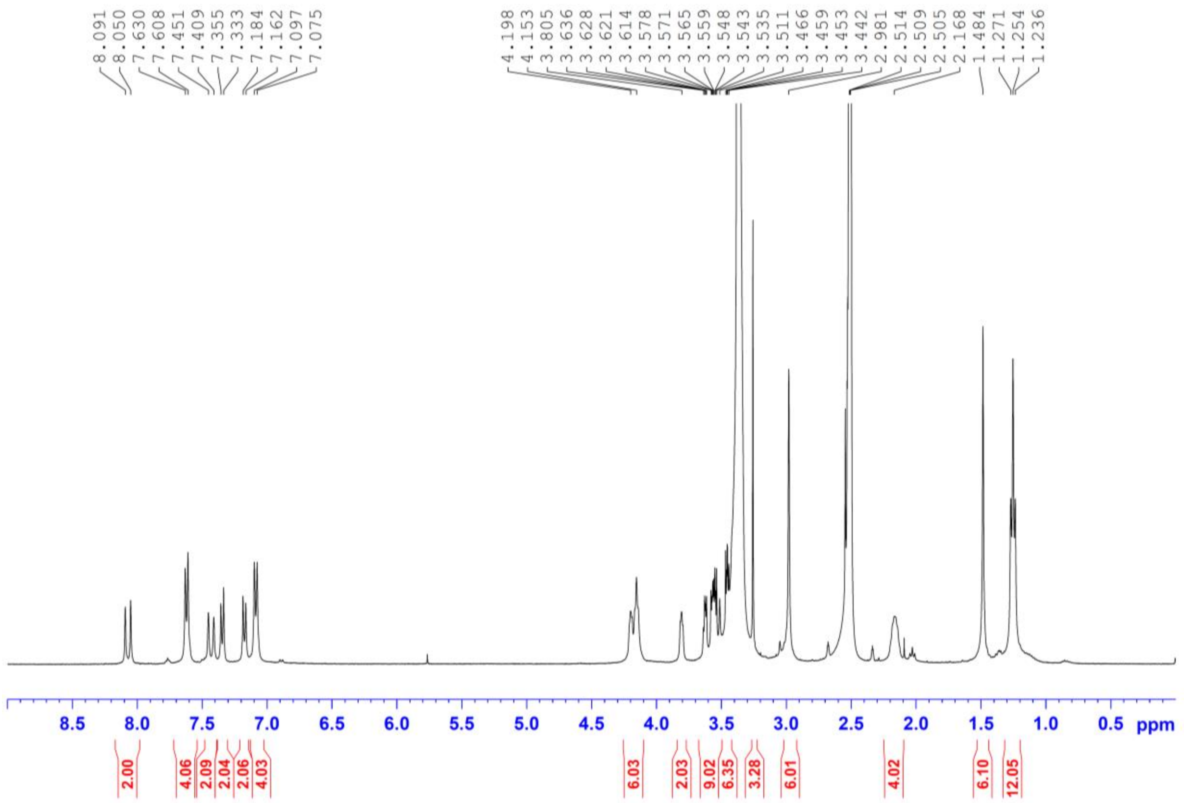

Figure S4. ^1H NMR spectrum of 4 in DMSO-d_6.

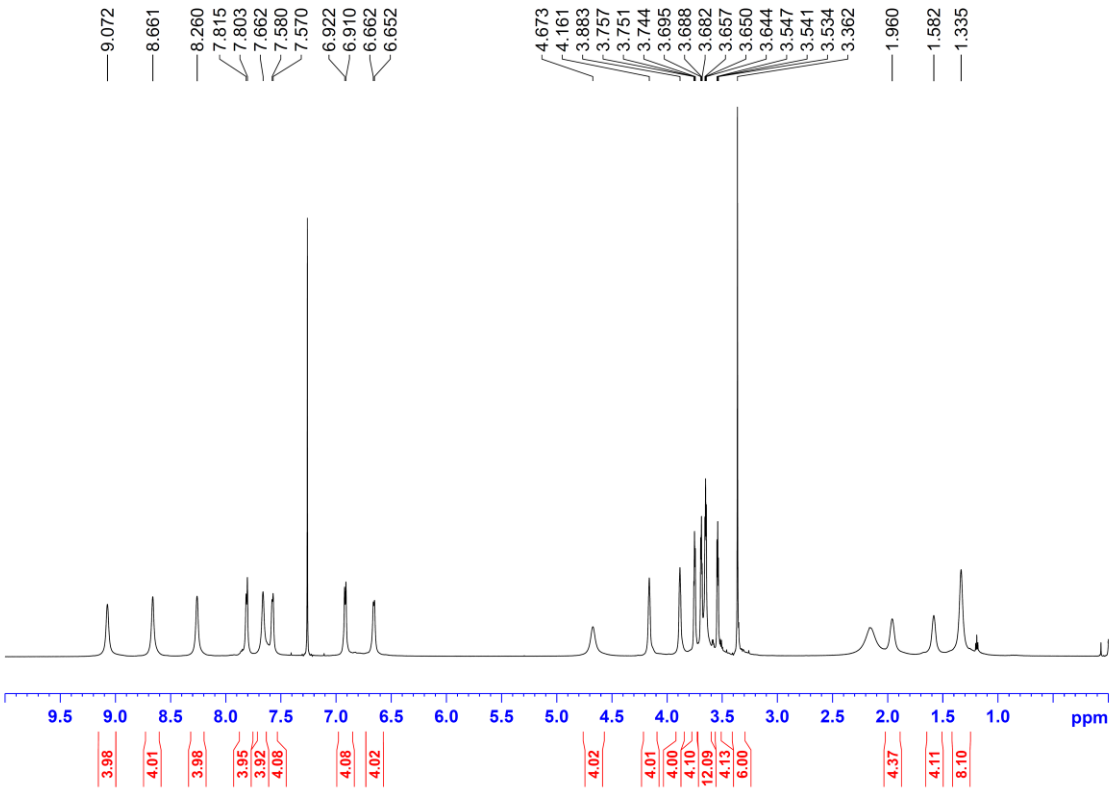

(**a**)

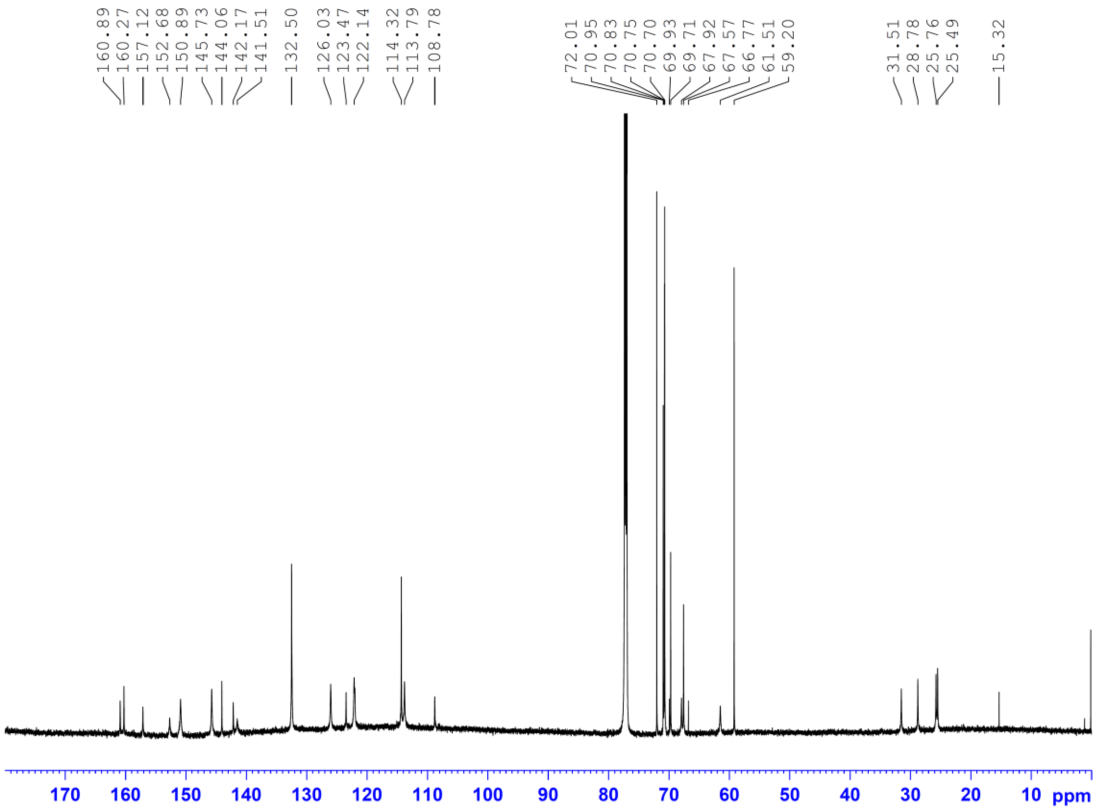

(**b**)

Figure S5. (**a**) ^{1}H and (**b**) $^{13}C\{^{1}H\}$ NMR spectra of 5 in $CDCl_3$.

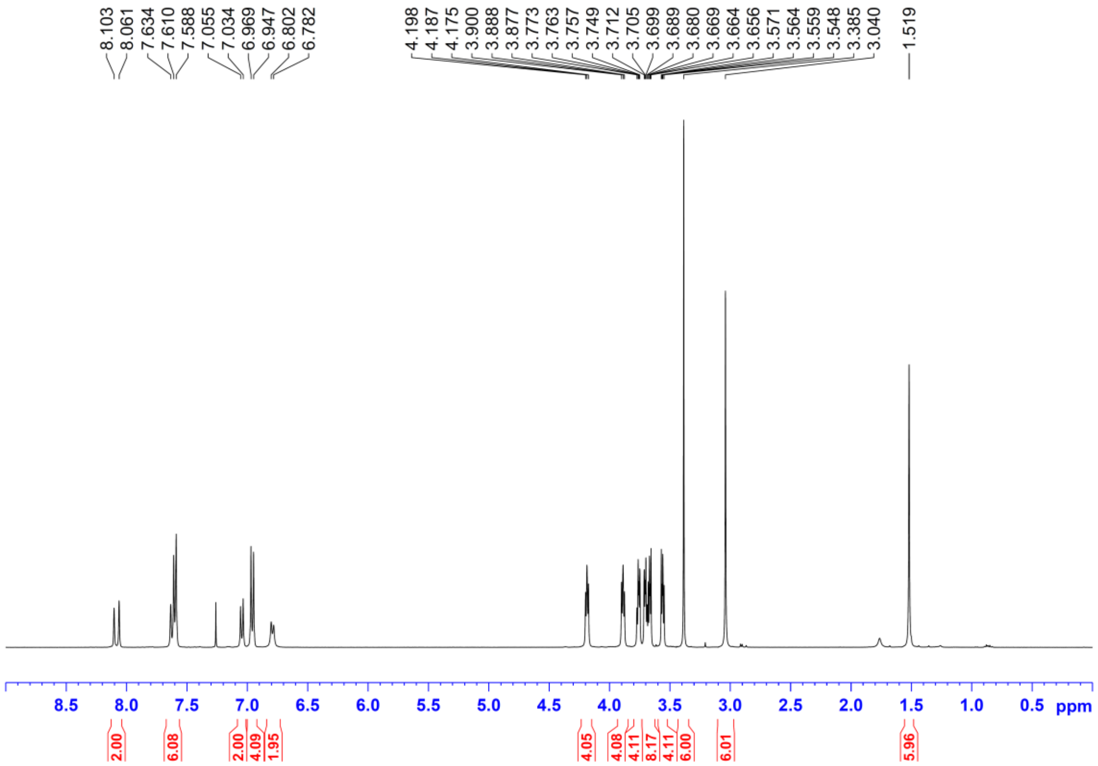

(**a**)

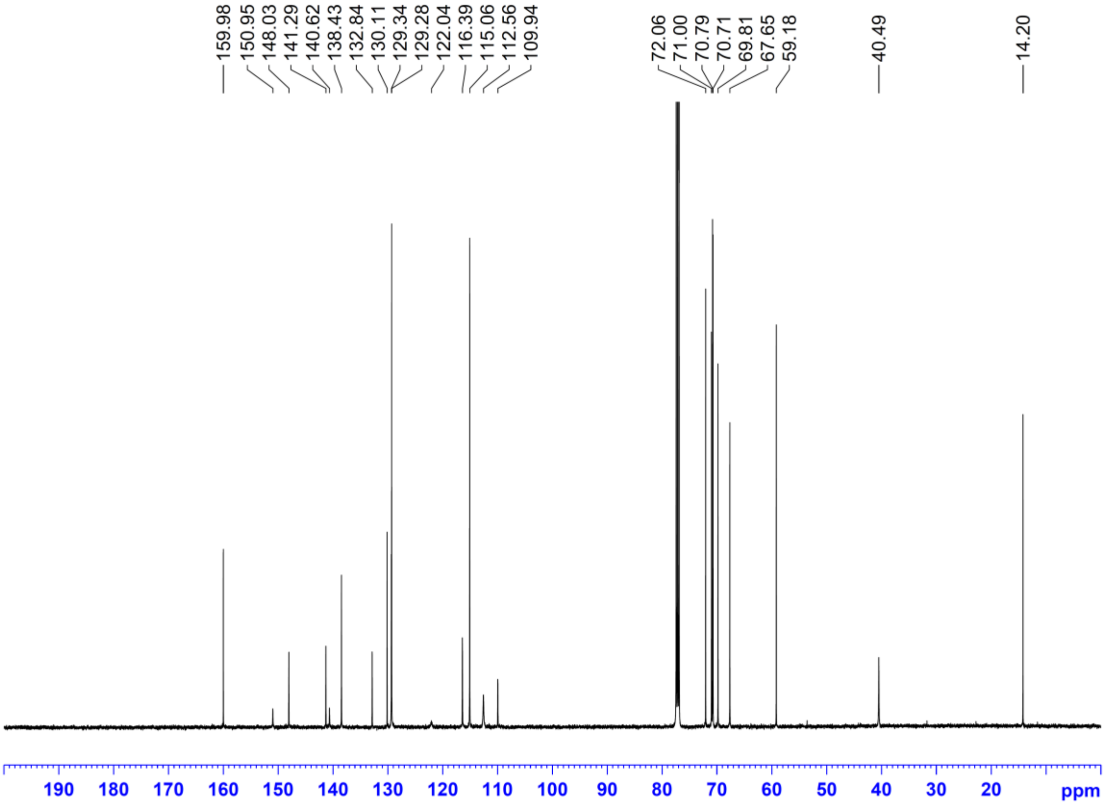

(**b**)

Figure S6. (**a**) ^{1}H and (**b**) $^{13}C\{^{1}H\}$ NMR spectra of 8 in $CDCl_3$.

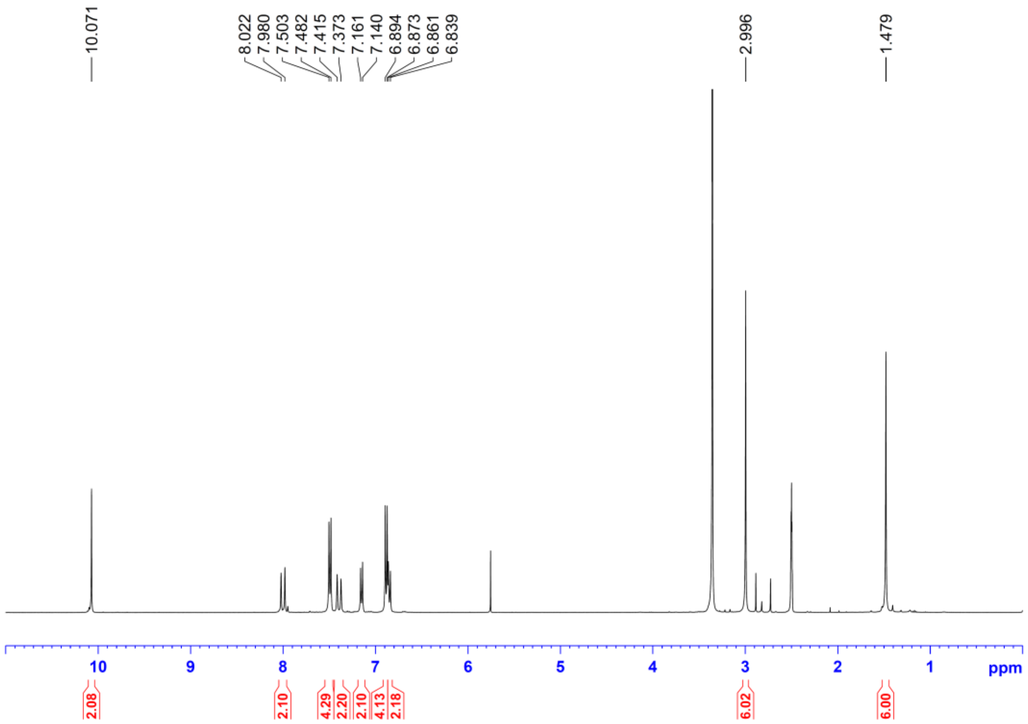

(**a**)

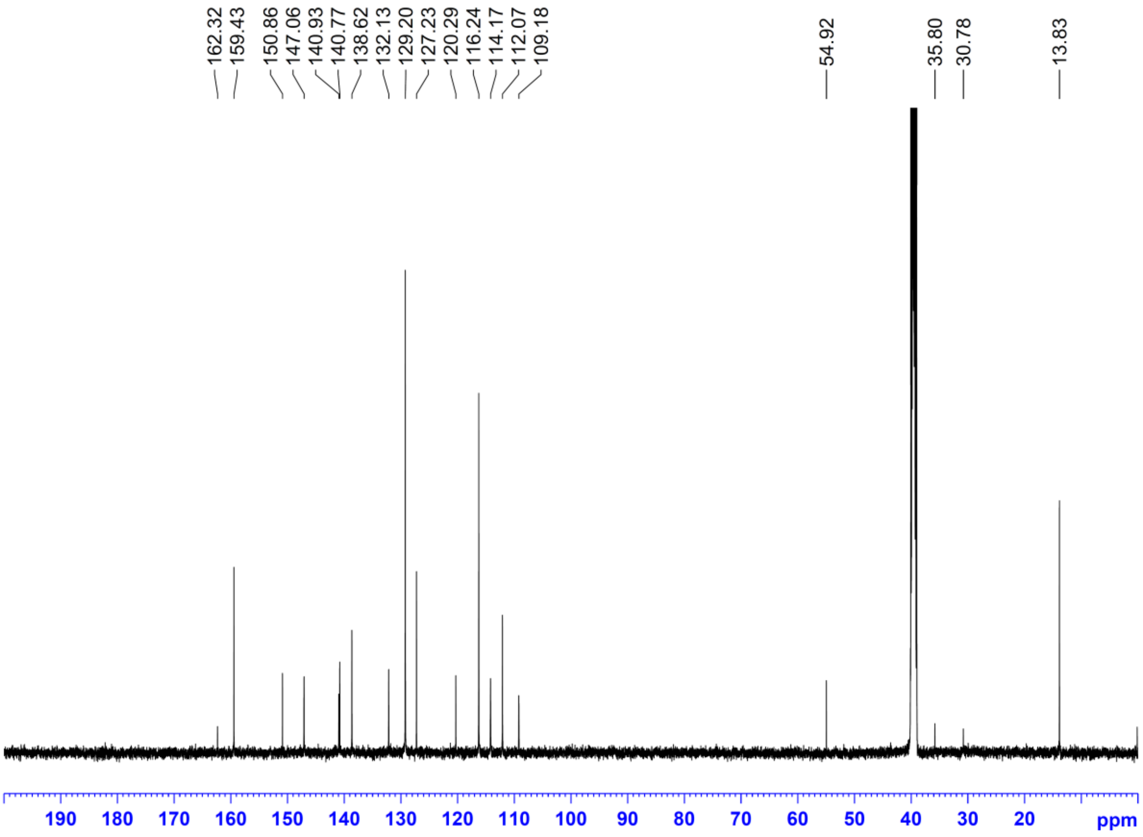

(**b**)

Figure S7. (**a**) ^{1}H and (**b**) $^{13}C\{^{1}H\}$ NMR spectra of 10 in DMSO-d_6.

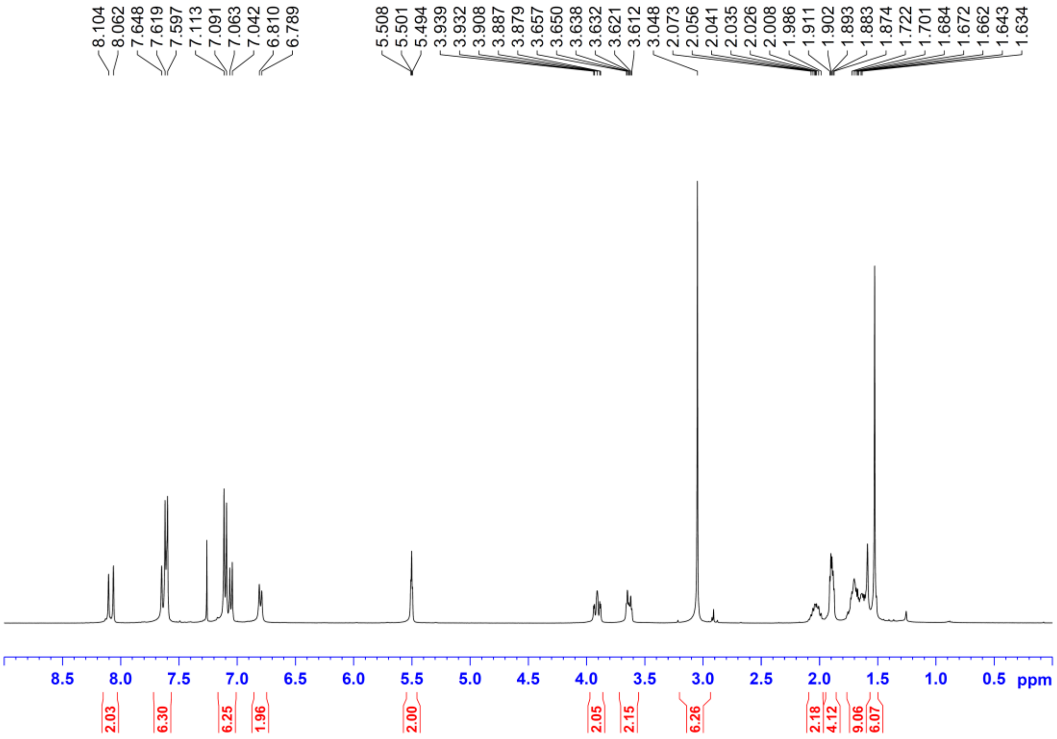

(**a**)

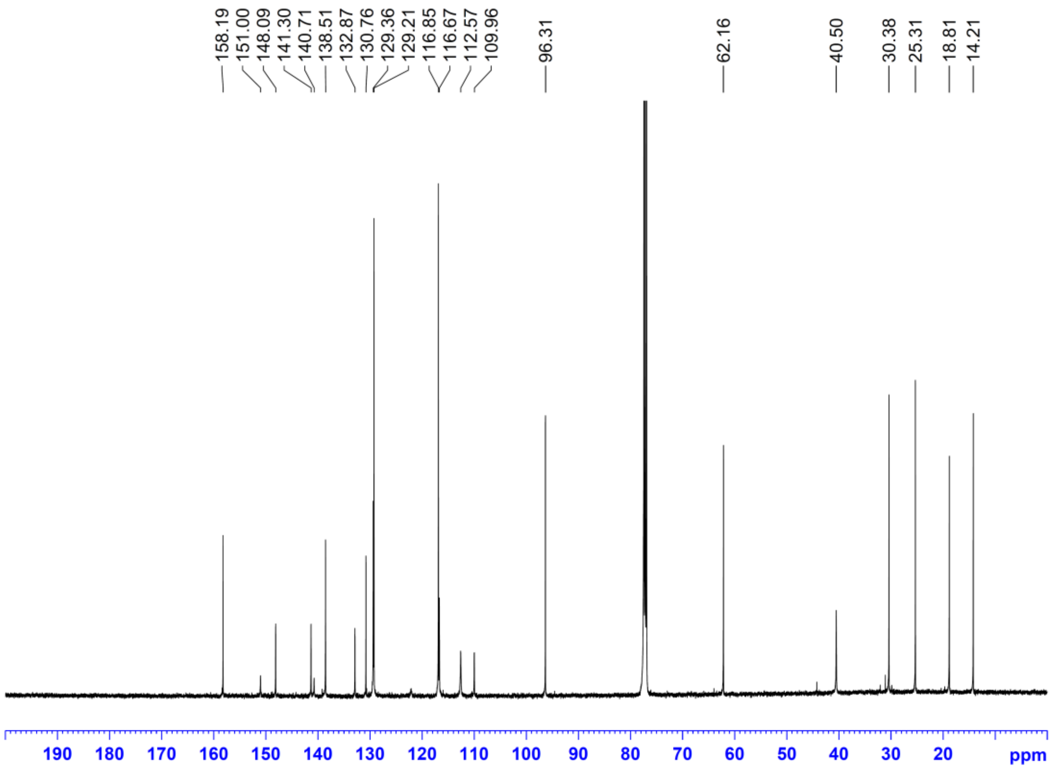

(**b**)

Figure S8. (**a**) ^{1}H and (**b**) $^{13}C\{^{1}H\}$ NMR spectra of 12 in $CDCl_3$.

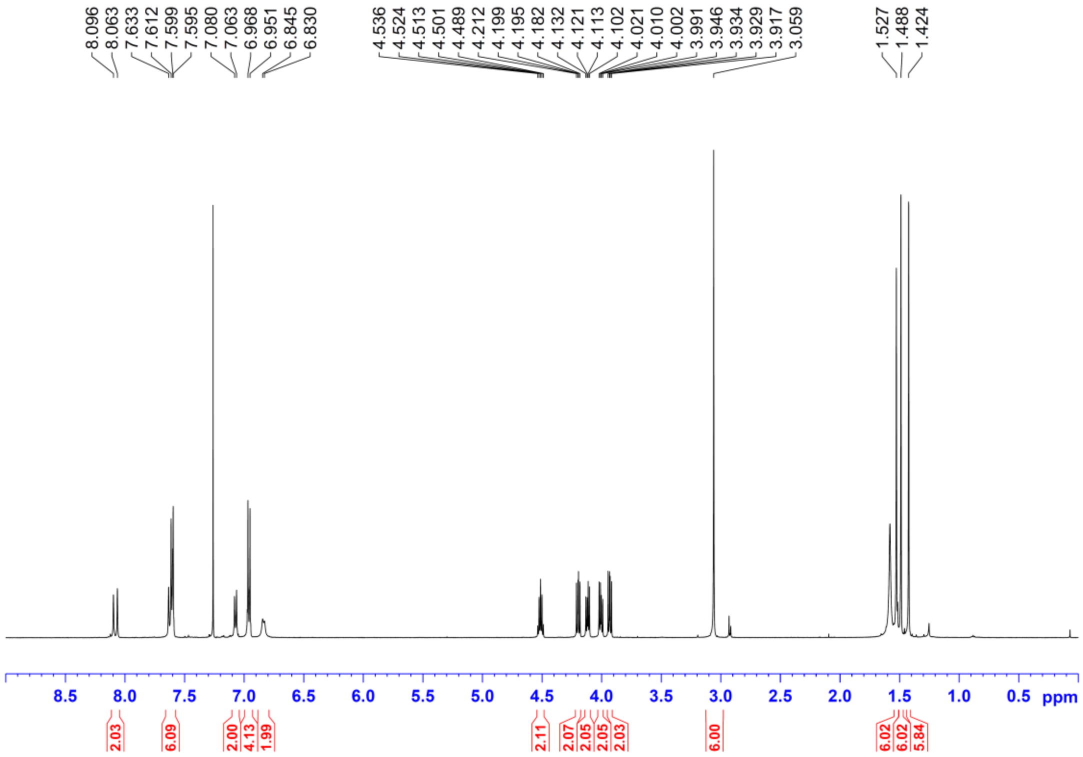

(**a**)

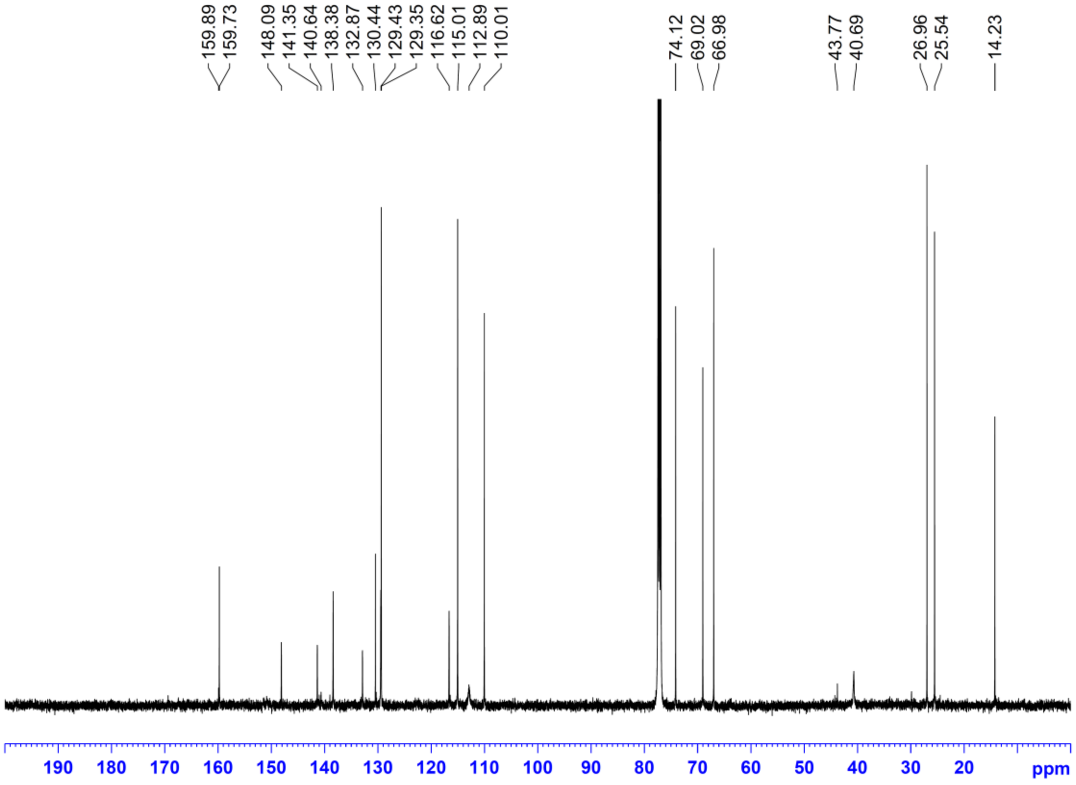

(**b**)

Figure S9. (**a**) ^{1}H and (**b**) $^{13}C\{^{1}H\}$ NMR spectra of 14 in $CDCl_3$.

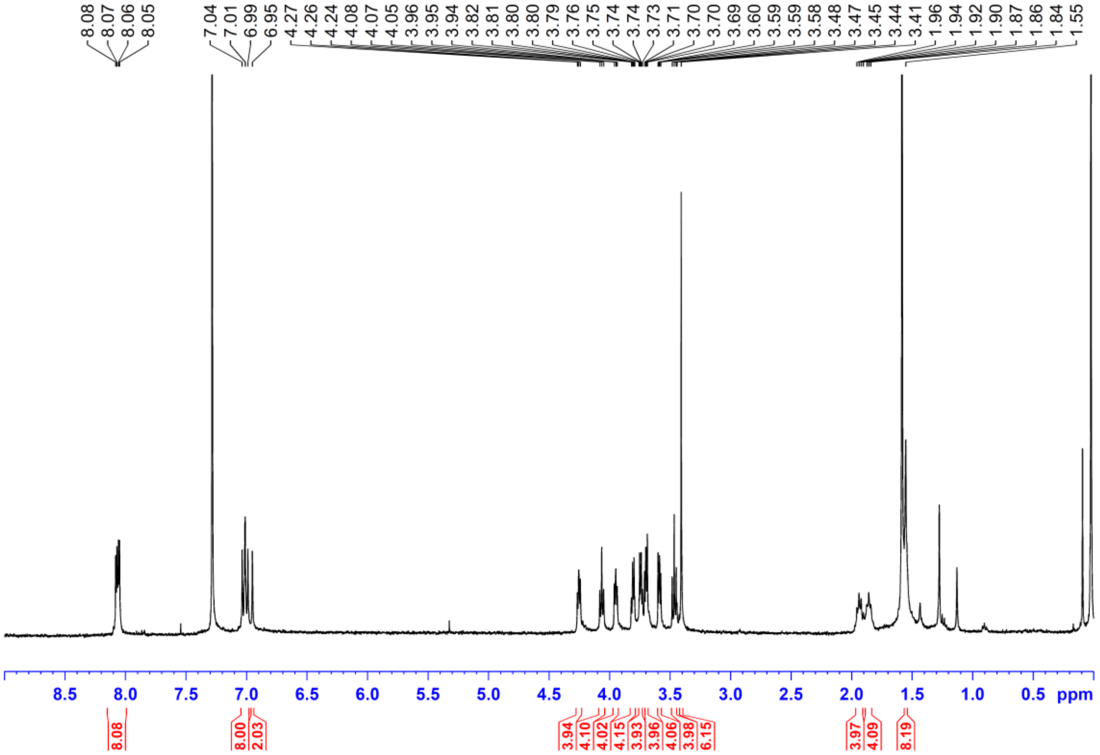

Figure S10. ^{1}H NMR spectrum of 18 in $CDCl_3$.

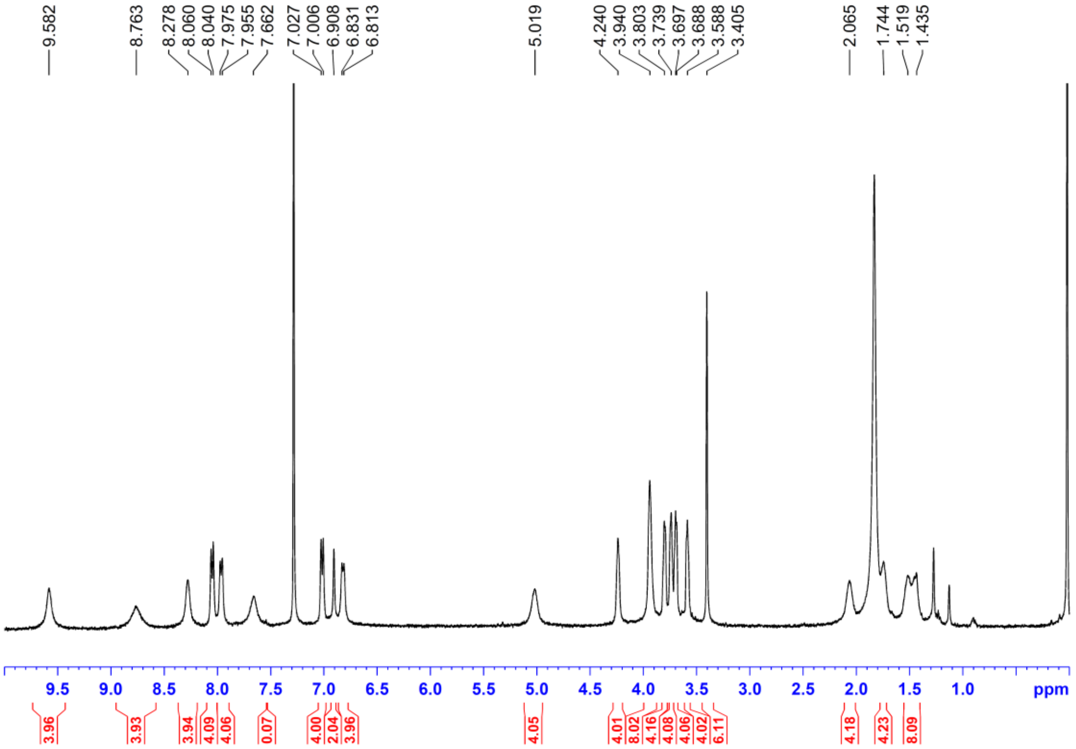

Figure S11. ^{1}H NMR spectrum of 19 in $CDCl_3$.
